# Long-term event-free and overall survival after risk-adapted melphalan and SCT for systemic light chain amyloidosis

**DOI:** 10.1038/leu.2016.229

**Published:** 2016-09-30

**Authors:** H Landau, M Smith, C Landry, J F Chou, S M Devlin, H Hassoun, C Bello, S Giralt, R L Comenzo

**Affiliations:** 1 Department of Medicine, New York–Presbyterian Hospital/Weill Cornell Medical College, New York, NY, USA; 2 Department of Medicine, Memorial Sloan Kettering Cancer Center, New York, NY, USA; 3 Department of Medicine, Icahn School of Medicine at Mount Sinai, New York, NY, USA; 4 Department of Medicine, Tufts Medical Center, Boston, MA, USA

## Abstract

Stem cell transplantation (SCT), an effective therapy for amyloid light chain (AL) amyloidosis patients, is associated with low treatment-related mortality (TRM) with appropriate patient selection and risk-adapted dosing of melphalan (RA-SCT). Consolidation after SCT increases hematologic complete response (CR) rates and may improve overall survival (OS) for patients with <CR. We retrospectively analyzed outcomes for 143 patients who underwent RA-SCT with or without consolidation. Melphalan was administered at 100 (14%), 140 (52%) and 200 (34%) mg/m^2^. The TRM rate at 100 days was 5%. RA-SCT resulted in CR in 24% (3 months) and 48% (12 months) of patients. The CR rate was particularly high (62%) in patients offered bortezomib consolidation. With a median follow-up among survivors of 7.7 years, median event-free survival (EFS) with RA-SCT was 4.04 years (95% confidence interval (CI): 3.41–5.01 years); median OS was 10.4 years (95% CI: 7.3–not achieved). Patients with CR at 12 months after SCT had significantly longer EFS (*P*=0.01) and OS (*P*=0.04). In a multivariate analysis, melphalan dose had no impact on EFS (*P*=0.26) or OS (*P*=0.11). For selected patients, RA-SCT was safe and was associated with extended long-term survival. With the availability of novel agents for consolidation, RA-SCT remains a very effective and important backbone treatment for AL amyloidosis.

## Introduction

High-dose melphalan plus autologous stem cell transplantation (SCT) is a standard treatment regimen for transplantation-eligible patients with systemic amyloid light chain (AL) amyloidosis. This regimen is based on initial reports that demonstrated high hematologic response rates, including complete hematologic remissions, frequent involved organ responses and extended overall survival (OS).^1^ A prospective randomized trial comparing high-dose therapy with standard dose melphalan and dexamethasone suggested that survival was better for patients who received oral chemotherapy than for those who underwent autologous SCT (56.9 vs 22.2 months; *P*=0.0004).^2^ These data must be interpreted with great caution, however, because of the 30% treatment-related mortality (TRM) rate reported,^2^ highlighting the need for careful selection of patients for SCT at specialized centers.

With the advent of novel agents, the role of SCT continues to be questioned, especially given safety concerns. Yet with appropriate patient selection and risk-adapted melphalan dosing, the TRM rate is low (4–10%).^3, 4, 5^ To maintain efficacy despite attenuated melphalan dosing, post-SCT consolidation with novel agents has been used. In a series of phase 2 trials, we safely administered thalidomide and dexamethasone, and then bortezomib and dexamethasone, after transplantation to treatment-naive patients with newly diagnosed AL amyloidosis.^3, 5^ Consolidation resulted in an increased frequency of hematologic complete response (CR), which has been associated with more significant organ responses and longer OS than reported in historical controls.^6^

We now report the long-term outcomes of patients with AL amyloidosis who underwent risk-adapted melphalan plus SCT (RA-SCT) over an 11-year period; the median length of follow-up among survivors was 7.7 years. We also report the impact of risk-adapted dosing of melphalan and post-transplantation consolidation on outcomes in this patient population.

## Materials and methods

All patients included in this study had biopsy-proven AL amyloidosis and underwent RA-SCT at Memorial Sloan Kettering Cancer Center from February 2000 to June 2011. Patients with involvement of >2 major organs, New York Heart Association class III or IV heart failure, critical arrhythmias (atrial and ventricular tachycardias that result in unstable hemodynamics), or cardiac syncope were not eligible for SCT. Patients who met criteria for symptomatic multiple myeloma (based on standard criteria) were not included.^7^ The institutional review board approved the data collection required for this study, which was conducted according to the Declaration of Helsinki. Clinical and treatment data were extracted from a prospectively maintained database; all patients consented to have their medical records reviewed.

Peripheral blood stem cells were collected by leukapheresis after mobilization using granulocyte–colony-stimulating factor, as previously reported.^5^ Patients were assigned a melphalan dose (100, 140 or 200 mg/m^2^) based on age, cardiac involvement, and renal compromise (defined by creatinine clearance ⩽50 ml/min).^1^ Specifically, melphalan dose was based on risk group and age as follows: For patients with no evidence of cardiac involvement and creatinine clearance ⩾51 ml/min, the dose of melphalan was 200 mg/m^2^ if the patient was 60 years of age or younger, 140 mg/m^2^ if the patient was 61–70 years of age, and 100 mg/m^2^ if the patient was 71 years of age or older. For patients with cardiac amyloid and/or creatinine clearance <51 ml/min, the dose of melphalan was 140 mg/m^2^ if the patient was 60 years of age or younger and 100 mg/m^2^ if the patient was 61–70 years of age. Hematologic response was assessed at 3 months after transplantation, and patients not achieving CR were offered consolidation therapy; those who achieved CR were observed. Before 2007, consolidation therapy was given for 9 months and included thalidomide 50 mg nightly, which was escalated to 200 mg nightly over 2 weeks (as tolerated) along with dexamethasone 20 mg/m^2^/day orally for a 4-day pulse with up to 3 pulses per month. In 2007 and later, consolidation therapy included six cycles of bortezomib and dexamethasone (BD). The first two cycles included bortezomib (1.3 mg/m^2^ intravenous) administered on days 1, 4, 8 and 11, with dexamethasone (20 mg oral) added on days 1, 2, 4, 5, 8, 9, 11 and 12; the last four cycles of bortezomib (1.3 mg/m^2^ intravenous) were administered on days 1, 8, 15 and 22, with dexamethasone (20 mg oral) added on days 1, 2, 8, 9, 15, 16, 22 and 23.

Organ involvement was defined for each patient by standard and updated criteria.^8, 9^ Patients were assigned a cardiac stage (Mayo I, II, II) based on the cardiac biomarkers brain natriuretic peptide (BNP) and troponin.^10, 11^ Conversion between BNP and NT-proBNP was as follows: log BNP=0.28+0.66 × log NT-ProBNP; 86 pg/ml was identified as the appropriate cutoff.^11^ Bone marrow plasma cells (BMPCs) on initial diagnostic bone marrow were determined by using the highest estimate from the aspirate or biopsy.

### Response assessment

Hematologic response was assessed at 3 and 12 months after RA-SCT according to the International Society of Amyloidosis criteria.^9^ CR was strictly defined and required negative findings on serum and urine immunofixation electrophoresis, normal serum FLC ratio, and <5% clonal plasma cells on bone marrow studies; partial response, stable disease, and disease progression were defined as previously described.^9, 12^

### Biostatistics

OS and event-free survival (EFS) were calculated from the initial date of transplantation until the time of death (for OS) or until the date of next treatment or date of death (for EFS), whichever occurred first. Recognizing that criteria for progression in AL amyloidosis have never been validated and that current consensus criteria are often problematic,^13^ the date of next therapy was selected to estimate EFS because that was thought to represent the most objective, albeit imperfect, data point available. OS and EFS were estimated by Kaplan–Meier methods. Log-rank test was used to determine whether survival functions differed by covariates of interest. Cumulative incidence functions were used to estimate the incidence of disease-related mortality, treating death from other causes as a competing event. Gray's test was used to assess whether cumulative incidence functions differed by covariates of interest. A 12-month landmark analysis was used to assess the association between treatment response (CR vs <CR) and OS and EFS. Patients who did not have a 12-month follow-up were excluded. Cox proportional hazards model was used to evaluate the association between the following clinical factors and OS and EFS: melphalan dose (100 vs 140 vs 200 mg/m^2^), age at transplantation, number of organs involved (0–1 vs >1),^1^ BNP (<86 vs ⩾86 pg/ml),^4, 11^ troponin (<0.1 vs ⩾0.1 ng/ml),^4^ Mayo cardiac stage (I vs II vs III),^4^ and baseline 24-h creatinine clearance (⩾50 vs <50 ml/min).^4^ Multivariate Cox proportional hazards model was used to evaluate the association of melphalan dosage on OS and EFS, adjusting for number of involved organs, Mayo cardiac stage and induction chemotherapy. Multivariate Cox proportional hazards model was also used to evaluate the independent association between BMPCs (⩽10% vs >10%)^14^ and OS, adjusting for treatment response at 12 months and Mayo cardiac stage. Covariates for adjustment in multivariate models were chosen based on factors that were associated with outcomes on univariate analyses and based on clinical relevance. All *P* values were based on two-sided statistical tests, with *P*<0.05 considered statistically significant. All analyses were performed using statistical packages SAS version 9.3 (SAS Institute, Cary, NC, USA) and R version 2.3.1 to compute the test statistics.

## Results

### Patient characteristics

One hundred forty-three patients (Table 1) were included in this study. Eighty-three of the patients were treated according to one of two prospective phase 2 protocols (ClinicalTrials.gov NCT01527032 and NCT00458822), and the remaining 60 patients were treated off protocol because either they were treated before the studies were available (before 2002) or they had received one or two cycles of initial therapy elsewhere. Their median age was 56 years (range: 49–62 years). Most patients were treated with 140 mg/m^2^ melphalan (*n*=74; 52%), but some (*n*=48; 34%) received 200 mg/m^2^ and others (*n*=21; 14%) received 100 mg/m^2^. Most patients had kidney involvement (*n*=92), followed by heart (*n*=58), gastrointestinal/liver (*n*=50), and nervous system (*n*=26) involvement. Half the patients had more than one involved organ. Cardiac biomarkers (BNP 0–3492 pg/ml, troponin 0–0.12 ng/ml), available for 100 patients, classified patients as having Mayo cardiac stages I (34% *n*=34), II (49% *n*=49) and III (17% *n*=17) disease. In 103 patients with available pretreatment creatinine and 24-h urinary protein values, 46% (*n*=47), 42% (*n*=43) and 13% (*n*=13) had stage I, II and III renal involvement, respectively.^15^ Before treatment, 63% (*n*=90) of diagnostic bone marrow aspirate and biopsy samples contained ⩽10% BMPCs, and 37% (*n*=53) contained >10%. Baseline characteristics by study protocol or off-label treatment are presented in Supplementary Table 1.

### Event-free survival and overall survival

**Figure 1:**
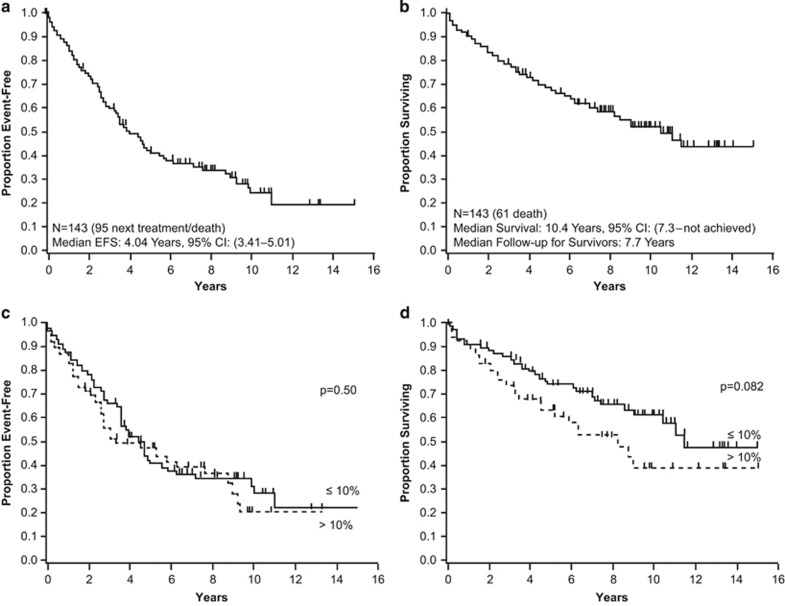
EFS and OS after RA-SCT and by BMPC burden at diagnosis. (**a**) Median EFS of 4.4 years was observed after RA-SCT. (**b**) Median OS after RA-SCT was 10.4 years. Patients with ⩽10% BMPCs had similar (**c**) EFS (*P*=0.50) and (**d**) a trend toward longer OS (*P*=0.08) than patients with a higher burden of plasma cell disease at baseline (>10% BMPCs).

With a median follow-up among survivors of 7.7 years, the median EFS in 143 patients who underwent RA-SCT was 4.04 years (95% confidence interval (CI): 3.41–5.01 years) (Figure 1a), and median OS was 10.4 years (95% CI: 7.3–not achieved) (Figure 1b). Eight patients died within 100 days of RA-SCT; 7 of them had advanced heart disease, accounting for 5% TRM rate. Among the patients who received melphalan 100 or 140 mg/m^2^, 2 and 6 patients, respectively, died within 100 days; there was no TRM in patients who received melphalan 200 mg/m^2^. Fifty-eight patients needed therapy after RA-SCT, and 30 patients died before the next treatment. Eighty-two (57%) patients remain alive at the end of follow-up.

**Figure 2:**
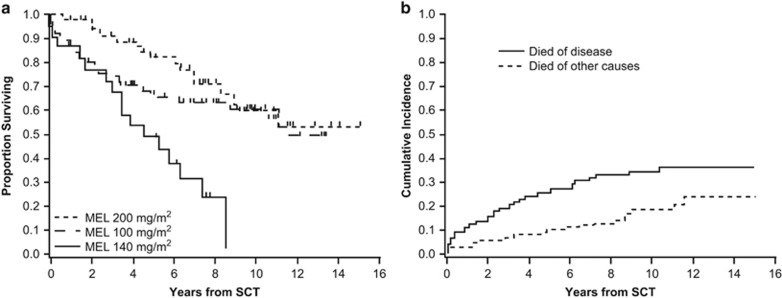
OS by melphalan dose and cumulative incidence of disease-related mortality. (**a**) The absence of patient deaths in the melphalan 200 mg/m^2^ group indicated that host factors warranting dose adjustment in patients with AL amyloidosis were responsible for early death. (**b**) The disease-related mortality rate 2 years after RA-SCT was 11%, and it increased to 24% at 5 years. The risk for death from AL amyloidosis appeared to stabilize 5 years after RA-SCT, whereas the risk for death from other causes continued to increase. MEL, melphalan.

Univariate and multivariate analyses for EFS and OS by patient and study characteristics are enumerated in Tables 2 and 3. By univariate analysis, melphalan dose (*P*=0.044), organ involvement (1 vs >1) (*P*<0.001), Mayo cardiac stage (I, II or III) (*P*=0.036), and induction of chemotherapy (*P*=0.004) were significantly associated with EFS. Similarly, melphalan dose (*P*=0.001), organ involvement (1 vs >1) (*P*=0.021), and Mayo cardiac stage (I, II, or III) (*P*=0.001) were associated with OS. Neither age at transplantation nor creatinine clearance was significantly associated with EFS or OS. There was no difference in EFS, but there was a trend toward better OS for patients with ⩽10% BMPCs at diagnosis (*P*=0.08) than for patients with a higher burden of plasma cell disease at baseline (>10% BMPCs) (Figures 1c and d). OS of the 143 patients based on melphalan dose are shown in Figure 2a.

By multivariate analysis, the effect of melphalan dose on both EFS (*P*=0.268) and OS (*P*=0.111) was no longer statistically significant when adjusted for number of involved organs, cardiac stage, and whether patients were previously treated with chemotherapy. The lack of TRM in the melphalan 200 mg/m^2^ group supports the view that patient and disease characteristics and comorbidities warranting risk-adapted dosing of melphalan in patients with AL amyloidosis are the basis for the TRM observed.

Given the ongoing risk for morbidity and mortality from organ disease, the long-term cause of death was evaluated (Figure 2b). At the time of this analysis, 61 of the 143 patients had died. Thirty-eight died of disease, and 23 died of other causes. The cumulative incidence of disease-related mortality 2 years after RA-SCT was 12% (95% CI: 6–17%) and increased to 24% (95% CI: 17–31%) at 5 years. The risk for death from AL amyloidosis appeared to stabilize 5 years after RA-SCT, whereas the risk for death from other causes continued to increase.

### Hematologic response

Hematologic response at 3 months and 12 months after RA-SCT was systematically assessed in 83 patients treated in clinical trials. Twenty patients (24%) achieved CR at 3 months after transplantation; this increased to 40 patients (48%) by 12 months after RA-SCT performed after consolidation (Table 4). CR frequency at 12 months after transplantation was particularly high (62%) in patients treated according to the protocol that offered bortezomib-based consolidation. CR frequency increased as well, from 21 to 36%, in the 44 patients offered thalidomide and dexamethasone after RA-SCT.

**Figure 3:**
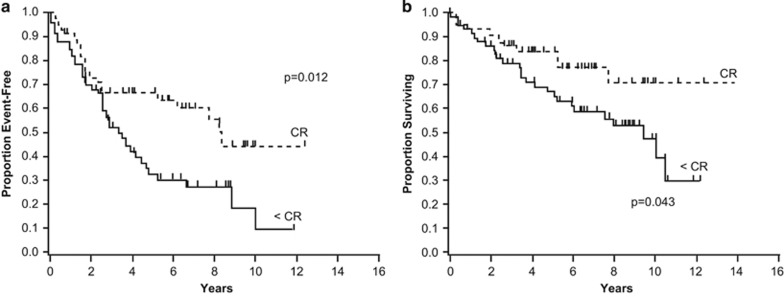
Landmark analysis results. Landmark analysis demonstrated that achieving complete response 1 year after RA-SCT was associated with superior (**a**) EFS (*P*=0.012) and (**b**) OS (*P*=0.043).

A landmark analysis was conducted to determine the effect of hematologic response on EFS and OS at 1 year after RA-SCT. Achieving CR 1 year after RA-SCT was associated with superior EFS (*P*=0.012) and OS (*P*=0.043) (Figure 3). However, there was no difference in either EFS or OS for patients who achieved CR at 1 year with consolidation compared with patients who achieved CR without consolidation (EFS, *P*=0.96; OS, *P*=0.24). Interestingly, after adjusting for treatment response at 1 year and cardiac stage, a higher burden of plasma cell disease at baseline was an independent risk factor for OS (HR 2.6; 95% CI: 1.1–6.1; *P*=0.036).

## Discussion

This retrospective analysis describes the long-term outcomes of 143 patients who underwent RA-SCT and posttransplantation consolidation at Memorial Sloan Kettering Cancer Center. In our analysis, RA-SCT was associated with low TRM and, compared with other national averages,^16, 17^ excellent EFS and OS. Consolidation after RA-SCT resulted in CR in one-third of patients who did not achieve CR with RA-SCT alone. Because CR is associated with prolonged disease control, carefully selected patients derived substantial therapeutic benefit from RA-SCT with consolidation. Moreover, this treatment approach is suitable as an initial therapy for patients with newly diagnosed AL amyloidosis.

Our data demonstrate that RA-SCT is safe, as do registry data that include 800 patients with AL amyloidosis who underwent transplantation between 2007 and 2012, and confirm that the 100-day mortality rate is 5%.^18^ TRM was attributed primarily to advanced cardiac involvement from AL amyloidosis, highlighting the need for early diagnosis and treatment of AL amyloidosis to improve OS. Although variables such as serum troponin >0.06 ng/ml, NT-proBNP >5000 pg/ml,^19^ and systolic blood pressure^20^ have been useful to identify subgroups of patients with poor prognosis, clinical criteria rather than cardiac biomarkers were used to select patients in the current analysis. We also confirmed that the association between Mayo cardiac stage and inferior outcome included EFS and OS. It is likely that in patients with disease of comparable severity undergoing initial therapy with regimens that do not involve transplantation, TRM would be similar.^21^ However, the duration of disease control may or may not be the same.

Consolidation after RA-SCT increased response rates and accounted for the similar OS among patients treated with 140 or 200 mg/m^2^ melphalan.^22^ Patients who achieved CR with RA-SCT alone or with RA-SCT plus consolidation fared equally well overall. As such, initial RA-SCT followed by consolidation only in patients who do not achieve CR is associated with excellent long-term survival and limits the up-front exposure to novel therapies. It is possible that reserving novel therapies for relapse after a disease-free interval will result in longer durations of second remission. The finding that greater plasma cell burden at diagnosis also affected OS in patients who received RA-SCT independently of cardiac disease, treatment response, and/or consolidation suggests that a more proliferative clone in AL amyloidosis may require multiple myeloma-like therapy and that these patients may benefit from induction therapy, prolonged maintenance therapy, or both. Cytogenetic abnormalities in patients with AL amyloidosis may help to further improve choice of therapy.^23^ With organ function at risk, minimal residual disease could also be important in guiding therapy after remission for patients with AL amyloidosis, but this has not been studied.

Historically, there has not been a role for induction chemotherapy in patients with AL amyloidosis.^24, 25^ Nevertheless, a recent small (*N*=56), single-center, randomized controlled trial comparing induction therapy with BD followed by SCT with SCT alone in patients with newly diagnosed AL amyloidosis showed improved outcomes that included the frequency of CR (*P*=0.03) and extended survival (*P*=0.03) in the group who received BD induction.^26^ Another trial,^27^ however, showed that despite high response rates to BD induction, the health of 14% of patients clinically deteriorated during initial therapy with BD, precluding SCT. Thus, determining the optimal timing of bortezomib-based therapy in association with RA-SCT and determining whether outcomes with RA-SCT are superior to those with proteasome inhibitor–based combinations alone in selected patients will require larger prospective, multicenter studies. The impact of consolidation, cytogenetic abnormalities, and minimal residual disease testing should also be evaluated prospectively in patients with AL amyloidosis.

In this report we show that in patients without advanced organ disease, initial RA-SCT was safe and was associated with excellent outcomes. Risk for early death was similar to or lower than that expected in this patient population.^17, 18^ The selection bias inherent in this data set must be recognized because AL amyloidosis patient eligibility for SCT is a favorable prognostic factor for survival.^28^ Missing data on treatment responses in our study confounds the analysis of EFS, which is another limitation. Yet the median EFS was 4 years, indicating the need for effective second-line therapy. Plasma cell–directed therapy—including MLN 9708, carfilzomib, pomalidomide and bendamustine—is under investigation. In addition, anti-amyloid therapies such as anti-serum amyloid P^29^ and NEOD001^30^ have the potential to improve organ function by reducing systemic amyloid deposits. A significant limitation of RA-SCT is its applicability to only a minority of patients with AL amyloidosis, estimated at approximately 25%, which highlights the need to diagnose the disease early before advanced organ damage develops.

At present, RA-SCT plus consolidation is among the most effective treatment strategies and should be considered for eligible patients. Although the current data are from a single institution, it is reassuring that autologous SCT resulted in a 5-year OS rate of 77% in a large, more contemporary cohort of selected patients reported to the Autologous Blood and Marrow Transplant Registry.^18^ To improve outcomes for more patients with AL amyloidosis, however, further education of physicians in the community is imperative.^31^ In this way, patients will benefit from earlier diagnosis and will be eligible for earlier effective treatment, including RA-SCT.

## Figures and Tables

**Table 1:** Demographics

*Characteristic*	*Patients* n=*143*
*Sex*	
Male	71
Female	72
Median (IQR) age at SCT, years	56 (49–62)
	
*Organ involvement*, n	
Renal	92
Cardiac	58
Gastrointestinal/liver	50
Nervous system	26
	
*No. organs involved*, n *(%)*	
1	72 (50)
>1	71 (50)
	
*Mayo cardiac disease stage*, n *(%)*	
I	34 (24)
II	49 (34)
III	17 (12)
Missing	43 (30)
	
*Renal stage*, n *(%)*	
I	47 (33)
II	43 (30)
III	13 (9)
Missing	40 (28)
	
*Melphalan dose, mg/m*^*2*^, n *(%)*	
100	21 (14)
140	74 (52)
200	48 (34)
	
*Induction of chemotherapy*, n *(%)*	
No	110 (77)
Yes	33 (23)
	
*Baseline measurements median (range)*	
CRCL	77 (0.06–193)
BNP	132 (0–3490)
TROP	0.06 (0–0.30)
BMPC	8.5% (1–53%)
	
*Cause of death*, n *(%)*	
DOD	38 (27)
DOO	12 (8)
DUK	7 (5)
DWD	4 (3)
Alive	82 (57)

Abbreviations: BMPC, bone marrow plasma cell percentage; BNP, brain natriuretic protein; CRCL, creatinine clearance; DOD, died of other disease; DOO, died of other causes; DUK, died of unknown cause; DWD, died with disease; IQR, interquartile range; RA-SCT, stem cell transplantation and risk-adapted dosing of melphalan; SCT, stem cell transplantation; TROP, troponin. Included are patients from two prospective phase 2 protocols (02–031 and 07-006) and patients treated off protocol who underwent RA-SCT from February 2000 to June 2011.

**Table 2:** Univariate and multivariate analyses of patient characteristics and treatments associated with EFS

*Characteristics*	*Univariate analysis*		*Multivariate analysis*	
	*HR (95% CI)*	P	*HR (95% CI)*	P
*Melphalan dose, mg/m*^*2*^		0.044		0.268
100	2.1 (1.1–3.7)		1.4 (0.6–3.2)	
140	1.2 (0.7–1.8)		0.8 (0.41–1.5)	
200	1.0		1.0	
*Age at SCT, years*	1.01 (0.9–1.03)	0.333		
*Induction of chemotherapy*		0.004		0.128
Yes	1.9 (1.2–3.0)		1.8 (0.8–4.1)	
No	1.0		1.0	
*No. organs involved*, n		<0.001		0.756
1	1.0		1.0	
2/3/4/5	1.8 (1.2–2.6)		1.1 (0.6–2.0)	
*Cardiac stage*		0.036		0.129
I	1.0		1.0	
II	1.8 (0.9–3.1)		1.7 (0.8–3.5)	
III	2.6 (1.2–5.5)		2.5 (1.02–6.1)	
*Baseline 24 h creatinine clearance, ml/min*		0.778		
⩾50	1.0			
<50	0.9 (0.5–1.5)			
*Baseline plasma cell*		0.501		
>10%	1.2 (0.8–1.7)			
⩽10%	1.0			
*Protocols*		0.316		
02–031	0.9 (0.5–1.4)			
07–006	0.7 (0.4–1.2)			
Off study	1.0			

Abbreviations: CI, confidence interval; EFS, event-free survival; HR, hazard ratio; SCT, stem cell transplantation.

**Table 3:** Univariate and multivariate analyses of patient characteristics and treatments associated with OS

*Characteristics/treatments*	*Univariate analysis*		*Multivariate analysis*	
	*HR (95% CI)*	P	*HR (95% CI)*	P
*Melphalan dose*, mg/m^2^		0.001		0.111
100	3.6 (1.7–7.2)		2.8 (0.9–8.5)	
140	1.4 (0.8–2.6)		1.4 (0.5–3.6)	
200	1.0		1.0	
*Age at SCT, years*	1.02 (0.9–1.05)	0.129		
				
*Induction of chemotherapy*		0.071		0.049
Yes	1.6 (0.9–2.8)		2.7 (1.0–7.6)	
No	1.0		1.0	
				
*No. organs involved*, n		0.021		0.477
1	1.0		1.0	
2/3/4/5	1.8 (1.1–3.0)		1.3 (0.6–3.0)	
				
*Cardiac stage*		0.001		0.022
I	1.0		1.0	
II	1.5 (0.6–3.6)		1.1 (0.4–2.8)	
III	5.0 (2–12.4)		3.5 (1.2–10.5)	
				
*Baseline 24-h creatinine clearance, ml/min*		0.805		
⩾50	1.0			
<50	0.9 (0.5–1.8)			
				
*Baseline plasma cell*		0.085		
>10%	1.6 (0.9–2.6)			
⩽10%	1.0			
*Protocol*		0.591		
02–031	0.9 (0.5–1.7)			
07–006	0.7 (0.3–1.4)			
Off study	1.0			

Abbreviations: CI, confidence interval; HR, hazard ratio; OS, overall survival; SCT, stem cell transplantation.

**Table 4:** Hematologic response at 3 and 12 months after RA-SCT in 83 patients

*Patients*	*Time after SCT*, n *(%)*	
	*3 months*	*12 months*
*All patients in phase 2 trials (*n=*83)*		
CR	20 (24)	40 (48)
Deceased/off study	4 (5)	10 (12)
		
*Patients achieving CR by consolidation*		
Post-SCT TD consolidation (*n*=44)	9 (21)	16 (36)
Post-SCT BD consolidation (*n*=39)	11 (28)	24 (62)

Abbreviations: BD, bortezomib and dexamethasone; CR, complete response; RA-SCT, stem cell transplantation and risk-adapted dosing of melphalan; SCT, stem cell transplantation; TD, thalidomide and dexamethasone.
