# Supplementary material for: Long-term event-free and overall survival after risk-adapted melphalan and SCT for systemic light chain amyloidosis
Source: Leukemia. 2016 Sep 30;31(1):136–42. doi: 10.1038/leu.2016.229 (PMC5220129; doi:10.1038/leu.2016.229)
Supplement: Supplementary Table S1 [file leu2016229x1.docx]

**Supplementary Table S1. Patient and disease characteristics by protocol or off-protocol treatment**

| **Characteristic** | **Protocol  02-031**  **n = 44** | **Protocol  07-006**  **n = 39** | **Off Protocol**  **n = 65** |
| --- | --- | --- | --- |
| Median age at  SCT, y (IQR) | 56 (49-64) | 57 (50-62) | 55 (49-61) |
| 100 | 5 (11.4) | 8 (20.5) | 8 (12.3) |
| 140 | 24 (54.5) | 17 (43.6) | 36 (55.4) |
| 200 | 15 (30.1) | 14 (35.9) | 21 (32.3) |

Numbers in parentheses are percentages unless otherwise indicated.

IQR, interquartile range; SCT, stem cell transplantation.
